# Psychosexual development and quality of life outcomes in females with congenital adrenal hyperplasia

**DOI:** 10.1186/s13633-015-0017-z

**Published:** 2015-10-15

**Authors:** Mansi Kanhere, John Fuqua, Richard Rink, Christopher Houk, David Mauger, Peter A. Lee

**Affiliations:** Department of Pediatric Endocrinology, Emory University School of Medicine, Atlanta, GA USA; Section of Pediatric Endocrinology, Riley Hospital for Children, Indianapolis, IN USA; Division of Pediatric Urology, Riley Hospital for Children, Indianapolis, IN USA; Department of Pediatrics, Georgia Regents University, College of Medicine, Augusta, GA USA; Public Health Sciences, Penn State Hershey Medical Center, Hershey, PA USA; Department of Pediatrics, Penn State College of Medicine, 500 University Drive, Hershey, PA 17033-0850 USA

## Abstract

**Background/Aims:**

Outcome information regarding females with classical congenital adrenal hyperplasia (CAH) have generally suggested poor quality of life (QoL), general maladjustment, problems regarding sexuality, and decreased fertility. The aim of this study was to assess QoL, psychosocial adaptation, and psychosexual characteristics, includingchildhood gender role behavior, gender identity, and sexual orientation in females with CAH.

**Methods:**

Female patients with 21-hydroxylase deficiency CAH were evaluated using a questionnaire with items relating to knowledge of their condition and its therapy; consistency of medical, surgical, and psychological care; childhood friends and play behavior; and genital, pubertal, and sexual development. The subjects’ perception of outcome was compared with family support and adolescent and adult QoL perspectives, including social relationships, self and body image, and gender and sexual issues.

**Results:**

Childhood play and gender characteristics, childhood and adult genital perception, and sexual identity and orientation varied as previously reported. However, most patients indicated good family support, understanding of their condition, good quality medical care, positive self-satisfaction, indices of happiness and body image perception, and satisfaction with their sex lives.

**Conclusion:**

The data reported here suggest that overall outcome can be very good for females with CAH and that good outcome appears to relate to quality of care and positive social support.

**Electronic supplementary material:**

The online version of this article (doi:10.1186/s13633-015-0017-z) contains supplementary material, which is available to authorized users.

The psychosocial outcomes of female patients with congenital adrenal hyperplasia (CAH) have not been consistently defined. Outcome information is limited regarding females who have the classical form of CAH, with reports of poor quality of life (QoL), general maladjustment, problems regarding sexuality, and decreased fertility [1, 2]. Among some, previous genital surgery was probably less refined than currently and may have resulted in external genitalia that impaired both sexual intercourse and sexual responsiveness. With refinements in medical therapy and surgery and changes in societal perspectives regarding gender and sexuality, QoL factors, including socialization and sexuality, need further assessment. Recent studies on QoL and psychosocial adjustment suggest less than satisfactory results; women with CAH have anxiety related to the appearance of their genitalia and decreased body image satisfaction leading to avoidance of sexual activity [3–5].

Exposure of the developing brain to androgens during fetal life is understood to impact post-natal gender and sexual development, including sexual orientation, although these relationships are both multifactorial and not predictable. Gender identity studies from 1950 to 2004 found that while females with CAH are more likely to exhibit male-typical childhood play, the majority who are raised female identify as female and live in their assigned gender [6]. Nevertheless, women with CAH have increased rates of non-heterosexual orientation as compared to the general population. The true frequency is unclear, since reported frequencies range from 3-31 % [7].

The impact upon QoL of factors such as family support, consistent medical care, and psychological counseling remain unverified. The hypothesis of this study was that family and other social and psychological support result in more positive perspectives during childhood and are related to factors suggesting better QoL during young adult life. To address these issues, herein we report results from a questionnaire for adult and adolescent females with CAH together with review of available medical and surgical history.

## Patients and methods

### Study population

Eighty-nine adolescents and adult women aged ≥14 were mailed questionnaires using the latest available address. Of these, 25 were undeliverable; of the 64 that were not returned, 37 did not respond, 27 responded with completed surveys. Data were coded by number, and only assessed as group data. Subjects were current or former patients treated by the Pediatric Endocrinology or Pediatric Urology Divisions at Riley Hospital for Children or former patients of an author (PAL) from the Children’s Hospital of Pittsburgh. Most were identified from medical records at Riley Hospital or the investigators’ research records, and a questionnaire was sent by mail. Research data were collected from the questionnaire. All had classic CAH (21-hydroxylase deficiency). Patients with additional diagnoses or chronic diseases were excluded.

Institutional review board approval was obtained at the Indiana University School of Medicine. Completion of the questionnaire was considered to be consent to participate.

### Methods

The specific aim of this study was comparison of responses to a questionnaire that was derived from general questionnaires in the public domain (Table 1), with items relating to patients’ knowledge of their condition and therapy; consistency of medical, surgical, and psychological care; childhood friends and play behavior; and genital, pubertal, and sexual development, perceptions, and experience. The participants indicated their age in years on the questionnaire as one of 4 groups: age 14 to 17, 18 to 21, 22 to 25, and ≥26. Their specific age was not available to us according to the IRB approval that considered the completion of the questionnaire as consent. Participants were allowed to omit answers to questions if they chose not to respond. Comparisons were made between the quality of surgical and medical therapy and family support vs. adolescent and adult QoL perspectives including social relationships, self and body image, and gender and sexual issues. The questionnaire consisted of 50 questionsl including closed-ended questions with a yes/no answer, nominal questions with graded responses from most favorable to least favorable, and open ended questions to allow subjects to express personal opinion. The questionnaire is avaliable as Additional file 1.

**Table 1:** Source references for questionnaire

1. Body Esteem Scale:S.L. Franzoi, S.A. Shields, (1984. *Journal of Personality Assessment* 1984; 48, 173–178.
2. Female Sexual Function Index (FSFI) R. Rosen, C Brown, Heiman J, Leiblum S, Meston C, Shabsigh R, Ferguson D, D’Agostino R Jr. *J Sex Marital Ther*. 2000;26(2):191–208
3. National Health and Social Life Survey (NHSLS Laumann EO, Gagnon JH, Michael RT, Michaels S. Chicago: University of Chicago Press, 1994.
4. Recalled Childhood Gender Identity Scale HF Meyer–Bahlburg, C Dolezal, KJ Zucker, SJKessler, JM Schober, MI New. J Sex Res. 2006;43:364–367
5. Sexual Behavior Assessment Schedule-Adult ((SBAS-A) de Souza Pinto D, CLV Filho, Wainberg ML, de Mattos PEL, Meyer-Bahlburg HFL, Rev. psiquiatr. Rio Gd. Sul vol.29 o.2 Porto Alegre 2007 http://dx.doi.org/10.1590/S0101-81082007000200012
6. SF-36 (www.sf-36-org)
7. The Gender Identity/Gender Dysphoria Questionnaire for Adolescents and Adults JJ Deogracias, LL Johnson, Meyer-Bahlburg HF, Kessler SJ, Schober JM, Zucker KJ. Sex Res. 2007; 44:370–379. doi:10.1080/00224490701586730.
8. The Masculine Gender Identity Scale for Females Blanchard R, Freund K, Journal of Consulting and Clinical Psychology,1983; 51: 205–214. http://dx.doi.org/10.1037/0022-006X.51.2.205
9. The Sex Role Behavior Scale-Orlofsky JL1, O’Heron CA. Orrlafsky and O’Heron J Pers Assess. 1987; 51:267–77.

The initial goal of the protocol was to compare severity of virilization at birth, genital surgery, and indices of glucocorticoid suppression with outcome indices. However, for the majority of study subjects adequate medical and surgical information was unavailable. Hence, statistical consultation (DM) recommended that responses be grouped to answer hypothesis-driven questions. These were as follows: to determine whether there was a direct relationship(s) between a) positive family support and positive self-esteem, b) recalled childhood and adult genital perceptions, c) understanding their condition, long-term physician relationship, and compliance, d) childhood gender view and behavior (male-type) and adult perceptions (wanting to be a man, preferred sexual partner), e) level of education, perceived knowledge of condition, family support, medication adherence, and adult social and sexual relationships and f) adult self perception, social relationships and sexual activities.

### Statistical analysis

Statistical analysis included descriptive statistics (mean, standard deviation, median and range of scores for each question). Fisher’s Exact Test was used to compare outcome variables related to socialization, gender, and sexuality.

## Results

### Demographics and diagnosis understanding

Table 2 outlines the demographic characteristics of the subjects, including age category at the time of the study, living conditions, perception of family income, level of education, and understanding of diagnosis. Based upon available records, 17/27 (63 %) of the total subjects were considered to be salt-losers. Based upon the limited information available, the majority were assumed to have mildly to moderately virilized genitalia. As outlined in Table 2, the majority of the women thought they knew enough about their diagnosis to care well for themselves, and only 1/27 (3 %) felt she understood the disease poorly.

**Table 2:** Demographic characteristics, understanding of diagnosis and family support

Age group, y	14–17	18–21	22–25	>26
*n* = 27	7	6	5	9
Living conditions, no. (%)	Overcrowded urban area	Rural area	Many close neighbors	
*n* = 27	1 (4)	6 (22)	20 (74)	
Perception of family income, no. (%)	Less than average	Comparable	Higher than average	
*n* = 24	3 (11)	15 (56)	6 (22)	
Level of education, no. (%)	Less than High School	Graduated High School	Some College	Completed College and beyond
*n* = 27	6 (22)	9 (33)	5 (18)	7 (25)
Understanding of diagnosis, no. (%)	Very well	Fairly well	Adequate	Poorly
*n* = 27	7 (25)	9 (33)	10 (37)	1 (3)
Person who helped with understanding diagnosis, no. (%)	Parents	Medical Doctor	Both parents and Medical doctor	Parents, Medical Doctor and Surgeon
*n* = 23	1 (4)	1 (4)	15 (65)	6 (26)
Family support	Very supportive	Uncomfortable discussing it	Avoid discussing it	Not supportive
*n* = 23	19 (82)	2 (9)	1 (4)	1 (4)

### Family support

Eighty-two percent of women felt their families had been very supportive. All 9 who thought that one family member was more supportive indicated their mother, with 3 including their siblings and 1 including her father. Four of the 26 women (15 %) thought one family member was less supportive, 1 naming her mother and the other their father, siblings, and both. The attitudes of the non-supportive member regarding their condition was described by 2 subjects as embarrassment and shame, while one reported an acceptance of her condition. Twelve of 27 (44 %) women had a sibling or siblings with CAH; 4 were male, 5 female, and 3 with both. Seven of these (58 %) thought having a sibling helped in their care; including 4 who had a male sibling and 3 who had siblings of both sexes. Of the 5 (42 %) who felt having a sibling did not make a difference, 4 had female siblings and 1 had a male sibling.

### Duration of care by endocrinologists, compliance, and medication side effects

**Fig. 1:**
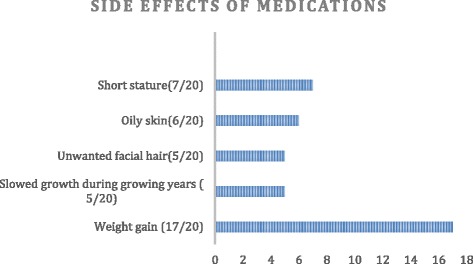
Side effects of medications as reported by the subjects

Table 3 depicts duration of care by endocrinologists. One person noted she had not had any care since loss of her parents’ insurance coverage at age 23 years. Frequency of compliance with medications is also shown in Table 3. Compliance was poorest during adolescence. Twenty-three of 24 (96 %) were responsible for taking their own medications. 16/23 (70 %) reported treatment compliance was the same since taking responsibility, 3 of 22 (13 %) said it was better and 3/22 (13 %) said it was worse. Figure 1 depicts side effects of medications. Other less frequently reported side effects were poor bone strength, stretch marks, and acne.

**Table 3:** Duration of care by endocrinologist and compliance with medications

Duration of care by endocrinologist, no. (%)	Long as I remember	At least 10 years	Between 5 - 10 years	<5 years
*n* = 24	15 (62)	2 (25)	3 (12)	4 (16)
Compliance with medications, no. (%)	Most of the time	Usually compliant	Never	
*n* = 27	23 (92)	3 (11)	1 (4)	
Age of most non-compliance, no. (%)	Childhood	Adolescence	Never missed medication	
*n* = 22	6 (26)	12 (56)	4 (17)

### Self-satisfaction

**Fig. 2:**
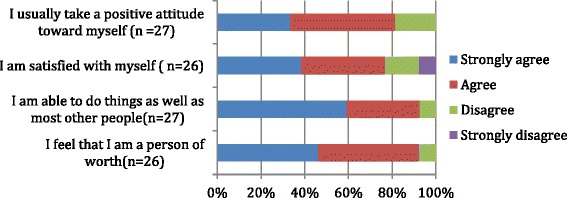
Questionnaire results: self worth and self satisfaction

Figure 2 shows the percentage of women who strongly agreed/agreed with having positive attitudes, being self-satisfied, self-competent regarding their abilities, and with having feelings of self-worth.

### Body image perception

Selected body image perceptions were: 19/23 (83 %) wanting to be taller, and 18 (78 %) wanting to weigh less. Eight women (35 %) wanted larger breasts, 6 (26 %) wanted different genital appearances, 4 (17 %) thought they had too much facial hair, and 4 (17 %) thought they had too much body hair.

### Physical pubertal development

**Fig. 3:**
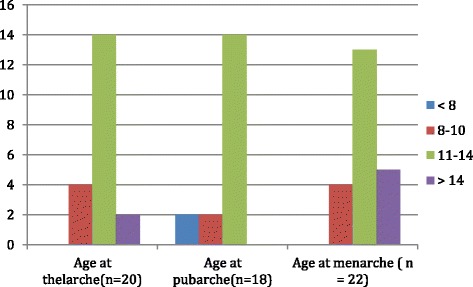
Age of onset of pubertal milestones

Figure 3 depicts age of onset of pubertal milestones. The age at thelarche and pubarche was late for most, reported between 11 and 14 years for 70 %; two women (10 %) indicated it had not yet occurred. Thirteen of 22 (59 %) had attained menarche between 9 and 13 years of age, and 6 reached menarche between ages 14 and 17 years. All except 3 (19/22, 86 %) reported having regular menstrual cycles.

### Childhood play and gender characteristics

**Fig. 4:**
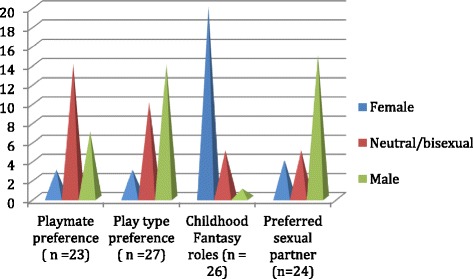
Declared adult sexual orientation identified as toward females, neutral/bisexual, or male compared with recalled childhood play preferences

Figure 4 depicts childhood play preferences and adult sexual orientation. Of 23 subjects who answered the question, 3 (13 %) preferred female playmates, 14 (60 %) were neutral, and 7 (30 %) preferred male playmates; data suggest these results were confounded by gender of siblings. Subjects were asked to select all applicable play preferences. Male-type play preferences were defined as toy cars/planes, sports, and rough and tumble play, and female-type play was defined as dolls/play houses and dressing up. Similarly, recalled childhood fantasy roles are shown in Fig. 4. The relationship between childhood fantasy roles and gender identity as an adult was statistically significant (*p* = 0.003). Those who assumed only female roles never experienced a desire to be a man. Of the 9 who assumed mostly female roles, 8 reported never wanting to be a man, with one (11 %) reported feeling this occasionally. Among the 5 girls who reported gender neutral fantasy roles, 2 indicated that they never wanted to be a man whereas 3 reported that this occurred occasionally. The one subject who preferred masculine fantasy roles as a child reported an occasional desire to be a man.

### Self-image, physique perception and happiness index

**Fig. 5:**
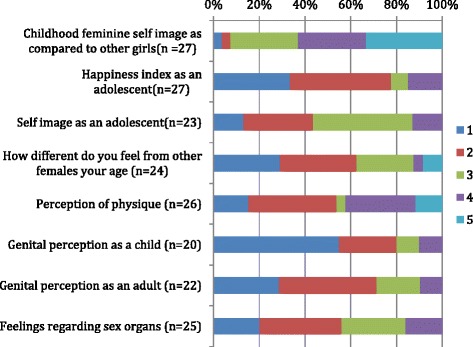
Self-image, physique and genital perception (Graded responses with best outcome = 1; worst outcome = 5). The percentage of women who picked each response is depicted in the figure

Figure 5 depicts the femininity, self-image, and physique perception as a child, in later stages of adolescence, and as an adult. These were reported as graded responses on a scale of 1–5, with 1 as best and 5 as worst. The percentages of women who picked each response are depicted in the figure. Compared to other girls during their childhood, only 2 of 27 (7 %) women felt more feminine, 8 (30 %) were about the same, and 17 (63 %) felt less feminine. Regarding a happiness index (“being happy about the kind of person you are”) as an adolescent, among 27 responders 21 (77 %) reported generally being happy, 2 (8 %) being indifferent, and 4 (15 %) being mostly unhappy. Self-image as an adolescent was reported by 10 of 23 (43 %) as positive and 10 of 23 (43 %) as ambivalent, with negative responses by 3 (13 %). Perception of physique in 26 patients was moderate to strongly positive for 14 (54 %), indifferent for 1 (3.8 %), and moderate to strongly negative feelings for 11 (42 %). Among those who had a positive physique perception, 82 % (9/11) report having had intercourse with a male; the other 2 had not. However, among those who reported a negative perception, 7 of 10 had had intercourse with a male and 3 had not had not (*p* = 0.004).

### Genital perception as a child versus as an adult

Among 20 patients, genital perceptions during childhood were perceived as like those of other girls for 11 (55 %), as different but acceptable for 5 (25 %), and as unacceptable for 4 (20 %). Current genital self-image among 23 patients was positive in 12 (52 %), acceptable for 7 (30 %) who indicated that they wished they looked better, and 4 (17 %) were unhappy with their genital appearance.

Comparing childhood and adult perceptions, 9 of 11 (81 %) women who thought their genitals appeared similar to those of other girls in childhood were happy with their appearance in adulthood; the remaining 18 % (2/11) wished they looked different. Of the 9 women who thought their genitals were different in childhood, only 1 (11 %) was happy with the appearance in adulthood and 8 (89 %) were unhappy with their appearance and wished they looked better (*p* = 0.002).

### Social and sexual relationships

The majority of women (21 of 24, 87.5 %) thought their condition did not interfere with social relationships, while 3 of 24 (12.5 %) indicated that it did. All 3 attributed this to “feeling different” and one to lack of confidence and the way people responded to her. A smaller majority (13 of 22, 59 %) felt that their condition did not interfere with their sexual relationships, with the remaining 9 (41 %) indicating that it did. These 9 attributed it to “feeling different” and “being embarrassed about their body”. One woman indicated it was because she was homosexual and afraid to reveal this and another because she only recently started to use dilators.

When comparing self-image relative to peers with feelings of loneliness, 3 of 7 of those who saw themselves as not different at all from peers reported no feelings of loneliness, and 4 reported feeling lonely occasionally. Among those who saw themselves as a little different, 7 % (1/14) reported no loneliness, 57 % (8/14) reported feeling lonely a few times in the last month, 7 % (1/14) felt lonely half the time, and 28 % (4/14) felt lonely most of the time in the last month. For the women who saw themselves as very different from their peers, 1 of 3 reported feeling lonely half the time and 2 of 3 most of the time (*p* = 0.06).

### Sexual identity and orientation

Sexual identity reported as frequency of desire to be a man, sexual orientation, and age of first intercourse are shown in Table 4. Seventeen of 25 (68 %) had achieved one or more of the sexual development milestones with males, including oral sex and/or sexual intercourse, 2 had never had intercourse but had been involved with kissing, and 6 (24 %) reported none. Thirteen of 22 (59 %) reported no physical involvement with females, 8/22 (36 %) women had some involvement including kissing, fondling, petting and oral sex, and 3/22 (14 %) had had genital to genital contact.

**Table 4:** Sexual identity and orientation

Desire to be a man, no. (%)	Never	Occasionally	Always
*n* = 24	18 (75)	6 (25)	0
Preferred sexual partner, no. (%)	Only/mostly male	Either males or females	Only/mostly female
*n* = 24	15 (62)	5 (21)	4 (16)
Age of first intercourse, no. (%)	>18	14–17	<14
*n* = 15	9 (60)	6 (40)	0

The relationship between childhood fantasy roles and gender of preferred sexual partner was statistically significant (*p* = 0.02). Seven of 8 women (87 %) who reported having only female fantasy roles preferred only male partners, while the other one preferred mostly male partners. Among patients indicating mostly female fantasy roles, 40 % (4/10) preferred only male sexual partners, 10 % (1/10) preferred mostly male partners, 30 % (3/10) were bisexual, and 20 % (2/10) preferred only female partners. Of those expressing neutral childhood fantasy roles, 20 % (1/5) preferred only male sexual partners, 20 % (1/5) preferred mostly male partners, 40 % (2/5) were bisexual, and 20 % (2/10) preferred mostly female partners. The one who preferred masculine fantasy roles as a child reported preferring only female partners.

### Satisfaction with sexual life

**Fig. 6:**
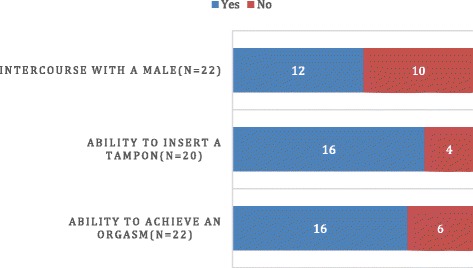
Sexual function

Figure 6 outlines issues related to sexual function: achievement of orgasm with stimulation of the clitoral/genital area, ability to insert a tampon, and achievement of intercourse with a male. Regarding sensations from stimulating the clitoris, on a scale of 1–5 with 1 being very good and 5 being poor, 77 % (14/18) reported them to be good, 23 % (4/18) thought they were adequate, and none reported them as poor. Of the 12 women who reported having sexual intercourse with a male, 6 (50 %) indicated difficulties; 5/12 (42 %) stated that it was painful. Four of the 10 (40 %) women who had never had intercourse with a male felt this was because they preferred females.

## Discussion

This limited report of factors that impact QoL suggests reasonable outcome for 46,XX patients born with moderate virilization due to CAH. The results are consistent with our hypothesis that family and other social and psychological support correlate with positive perspectives during childhood and better QoL during young adult life. These findings are consistent with reported studies relating to QoL. The statistical correlation between assuming a masculine fantasy role in childhood and both the desire to be male and a non-heterosexual orientation supports the theory by Hines et al. that recalled male-typical play in childhood correlates with reduced satisfaction with the female gender and reduced heterosexual interest in adulthood [8]. Previous reports have found poor satisfaction with body appearance, including genital appearance, while this report found a generally positive perception of physique that was statistically associated with having had sexual intercourse with males, consistent with recent reports [5, 9]. The QoL of most of the patients reported are reasonable compared with previously reported poorer QoL outcomes for adult women with CAH when compared with controls [10], albeit not associated with atypical gender role behavior [11]. When compared with a reference group, sexual function and functional outcome is less favorable [12]. Assessment of clitoral sensitivity among 10 sexually active patients with CAH was found to be significantly reduced compared with controls, but sexual function was not statistically or clinically significantly different. These data are consistent with good healthy sexual function among those in a healthy social situation even with diminished sensitivity [13].

Our findings of 68 % having had oral or genital intercourse or both with a male and 73 % having had an orgasm appears within the range of published reports [5, 12]. While many of our patients had not had intercourse with a male, this may be related to age and preference for female partners. In prior studies, the frequency of women who have experienced intercourse is generally low. Pain with intercourse was reported in 42 %, compared with previous reports ranging from 25 to 81 % [12, 14]. Although reported perceived sexual functioning was less satisfactory than reference groups [12], more than 80 % reported being able to achieve orgasm.

Most of our patients denied ever wishing to be male, and 60 % indicated sexual preference for males only, a minority having had sexual encounters with another female. Sixteen percent indicated sexual preferences for females, compared with 19 to 20 % of adult women with CAH reported in other studies [7, 14]. This study and a recent study did not identify any severe gender dysphoria, but incidences of 5 and 10 % have been reported [6, 11, 14]. Forty percent reported a sexual debut in the age group 14–17, earlier than a reported average age of 18.7 years [7]. Sexual activity was also more prevalent than previously reported, with 68 % sexually experienced with a male, and sexual involvement with females in 50 %. No involvement with a male was reported for 24 % of our subjects, compared to 37 % from a report of older women [14]. It should be noted that the age range in this study extended to younger individuals than most previous studies.

The broad range of pubertal milestones and menarche was comparable to that in the general population, in contrast to a previous report of early pubertal onset [10]. Most patients reported here saw the same endocrinologist most of their lives, indicated a reasonable understanding of their condition, and had reasonable compliance in contrast to reports from developing countries indicating inconsistent care and poor knowledge of the condition and treatment requirements [15].

Although detailed surgical information is not available for most of this study group and was inadequate for assessment, genital sensation was reported as good by 77 % of patients. Thus, most patients reported positive sensations during clitoral stimulation and the ability to achieve orgasm, compared with the findings in the literature of diminished clitoral sensitivity being worse in those with severe CYP21A2 mutations. Neither genetic analyses of mutations nor Prader stages at presentation were available in our patients, so severity of the enzyme defect cannot be assessed. A relationship has been reported between CYP21A2 genotype/severity of CAH/prenatal androgen exposure and psychological effects, including problems with gender identity and more surgical complications [1, 2, 5, 8, 12, 16, 17]. The former is evidenced by signs of gender and sexual orientation, with more severely affected women having an increased percentage of bisexual or homosexual orientation. Regarding surgical outcome, the higher the level of confluence of the urethra and vagina, the poorer the cosmetic outcome [12]. Since feminizing surgery and psychological adjustment is not satisfactory for those with the most severe virilization, it has been suggested that the dictum that all patients with a 46,XX karyotype be raised as females should be challenged and a male assignment should be considered those most virilized [18].

Other outcome data associated with severity of CAH include less formal education, more with disability pensions, sick leave, lower marriage rates, and fewer having children [19]. Previous reports have indicated poor academic performance and generally poor communication, which led to social limitations [20]. The current study found financial status and a spectrum of education similar to that of the general population, with no suggestion that their education level relates to the severity of their condition.

The limitations of this study include the lack of a control group; unavailability of complete medical records; and the lack of information about severity of virilization at birth, CYP21A2 genotype, and specific type of surgical procedures performed. Also, it must be noted that responses, particularly regarding sexual experiences, are those at the time of the questionnaire, and that broader experiences in the future may impact future QoL. When the number of undeliverable mailed questionnaires is subtracted from the total, the response rate was low (27/64, 42 %) likely due the length of the questionnaire, the lack of on-going contact of the authors with the patients, and the impersonal way (mail) the questionnaire had to be delivered. While the generalizability of our findings is limited by the small sample size, given the scarcity of outcome data on these important aspects of psychosexual health in females with CAH, the study contributes some valuable data.

## Conclusion

This report, which includes a majority of patients who had moderately virilized external genitalia at birth, suggests that outcome of CAH can be very good, with a relationship between both quality of care and positive social support and young adult QoL. Future outcome studies may produce more positive results, with refined surgical techniques, positive psychosocial support, and shifts in cultural acceptance having a positive impact [18, 19].

## Additional file

Additional file 1:
**Questionnaire.** (DOC 52 kb)
