# Supplementary material for: Psychosexual development and quality of life outcomes in females with congenital adrenal hyperplasia
Source: Int J Pediatr Endocrinol. 2015 Oct 15;2015:21. doi: 10.1186/s13633-015-0017-z (PMC4607144; doi:10.1186/s13633-015-0017-z)
Supplement: Additional file 1: — Questionnaire. (DOC 52 kb) [file 13633_2015_17_MOESM1_ESM.doc]

Subject Code Number:

You are being asked to complete this survey because you have been diagnosed with and treated for congenital adrenal hyperplasia (CAH). We would like to better understand how this diagnosis and its treatment have affected you and your loved ones. Please complete all items to the best of your ability by circling the answer or answers that best fit how you feel about the item. Please realize that there are no right or wrong answers!

Questions should be answered the best you can. If you prefer not to answer the question, you can indicate this. If you do not know the answer, you can leave it blank.

How old are you?

a. 14-17 years

b. 18-21 years

c. 22-25 years

d. 26 years or older

Where do you live?

a. A very crowded urban area

b. In an area with many close neighbors

c. In a rural area

Do you feel that your family has (more, the same, less) money that most people in

your country?

How many rooms in your home? ______

How many people sleep in the same room as you do?_____

How much education have you had?

a. Some schooling

b. Equivalent to high school

c. Some college

d. College degree

e. Advanced studies

These first questions relate to your congenital adrenal hyperplasia and how you were diagnosed and treated, how your family was involved and told about this:

1. How well do you feel you understand your diagnosis of congenital adrenal hyperplasia?
   1. Very well, I feel very comfortable with my knowledge level
   2. Fairly well, I feel good about my knowledge but would like to know more
   3. Adequate, I know enough to be able to care for myself
   4. Poorly, I feel like I understand very little about my diagnosis
   5. I don’t want to answer
2. Who has helped you to understand your condition? (Circle all that apply)
   1. Parents
   2. Medical Doctor
   3. Surgeon
   4. Counselor/Psychologist/Psychiatrist
   5. I don’t want to answer
3. How you have been informed about your condition? (Circle all that apply)
   1. It was explained to me over the years as I grew up.
   2. When I was an early teenager my doctor explained it to me.
   3. No one has ever really sat down and gone through the details with me.
   4. I have read about adrenal hyperplasia in books and handouts.
   5. I don’t want to answer
4. How supportive has your family been with helping you to understand your condition?
   1. Very supportive / easy to talk to / kept well informed.
   2. They tried but were uncomfortable discussing it.
   3. They did not seem willing to discuss it or seemed to avoid discussing it.
   4. They were not supportive and often made me feel sad about it.
   5. I don’t want to answer
5. Was one family member more understanding and supportive about your condition than other members? (Yes or No)

If so, who was this?

- 1. Mother
  2. Father
  3. Sibling (brother/sister)
  4. Other relative
  5. I don’t want to answer

1. Which statement(s) best describe the attitude of the person you indicated above? (Circle all that apply)
   1. Accepting of you and your condition.
   2. Open about sexual issues.
   3. Proud of you.
   4. Embarrassed about your condition.
   5. Ashamed of your condition.
   6. I don’t want to answer
2. Was one family less supportive or understanding about your condition? (yes/no)

If yes, who was this?

- 1. Mother
  2. Father
  3. Sibling (brother/sister)
  4. Other relative
  5. I don’t want to answer

1. If yes, which statement(s) best describes this family members attitude? (Circle all that apply)
   1. Accepting of you and your condition.
   2. Open about sexual issues.
   3. Proud of you.
   4. Embarrassed about your condition.
   5. Ashamed of your condition.
   6. I don’t want to answer
2. How many siblings do you have? (Circle all that apply)
   1. None
   2. One Sister
   3. One Brother
   4. Two or more Sisters
   5. Two or more Brothers
   6. I don’t want to answer
3. Do you have a sibling who has congenital adrenal hyperplasia? yes/no

If yes, is this sibling a boy or girl?

Has having a sibling been helpful for you in taking care of yourself? yes/no

1. How long has your endocrinology doctor cared for you?
   1. As long as I remember
   2. At least 10 years
   3. 5 to 10 years
   4. 2 to 5 years
   5. Less than 2 years
   6. I don’t want to answer
2. How often did you take your medications as prescribed?
   1. Most or all of the time
   2. Usually
   3. About half of the time
   4. Seldom or never
   5. I don’t want to answer
3. During which time(s) did you most miss taking medicines as prescribed? (Circle all that apply)
   1. Childhood
   2. Early Adolescence
   3. Late Adolescence
   4. All of these times
   5. I don’t want to answer
4. Which, if any, of the following problems did your medicines cause for you? (Circle all that apply)
   1. Weight gain
   2. Slow growth
   3. Unwanted facial hair
   4. Oily skin
   5. Rapid growth during childhood which finally stopped, leaving you short
   6. Other: Please tell us what this was. ______________________________
   7. I don’t want to answer
5. Do you think your condition interferes with your social relationships? yes/no If yes, why?____________________________________________________
6. Do you think your condition interferes with your sexual relationships? yes/no If yes, why?____________________________________________________
7. Are you responsible for taking your medications? yes/no

If yes, since what age? _____years

Is your treatment (better /the same / worse) since you started

being responsible for your own treatment?

The following section asks about how your body developed and what you think

about that and about your interests

1. Please circle each point that applies to you
   1. I would like to be taller
   2. I would like to weigh less
   3. I think I have too much hair on my body
   4. I wish my breasts were bigger
   5. I wish my genitals were different
   6. I think that I have too much hair on my face
2. Please indicate the age when each of the following started or indicate that it has not yet occurred
   1. Hair around my genitals Years of age_______Not yet_______
   2. Breast growth Years of age_______Not yet_______
   3. First periods Years of age_______Not yet_______
   4. Monthly periods Years of age_______Not yet_______
3. When you were a child, were your favorite playmates
   1. Always girls
   2. Mostly girls
   3. Either girls or boys
   4. Mostly boys
   5. Always boys
   6. I don’t want to answer
4. As a child, did you prefer to play with (circle all that apply).
   1. Toy cars/planes
   2. Dolls/play house
   3. Play sports
   4. Rough and tumble play
   5. Dressing up
   6. I don’t want to answer
5. As a child, I played
   1. Sports with girls
   2. With cosmetics/makeup
   3. Games with boys or girls
   4. With boy-type toys
   5. Sports with boys
   6. I don’t want to answer
6. As a child, compared to other girls my age I felt
   1. Much more feminine
   2. Somewhat more feminine
   3. About the same
   4. Somewhat less feminine
   5. Much less feminine
   6. I don’t want to answer
7. When I was a child, I fantasized or took the role of
   1. Only girls or women
   2. Mostly girls or women
   3. Both males and females
   4. Mostly boys or men
   5. Only boys or men
   6. I don’t want to answer
8. Now, as an adolescent or adult, how happy are you about the kind of person you are?
   1. Very happy
   2. Mostly happy
   3. Neither happy nor unhappy
   4. Mostly unhappy
   5. Very unhappy
   6. I don’t want to answer
9. As an adolescent or adult, how do you feel about your looks?
   1. Very good
   2. Pretty good
   3. No strong feelings either way
   4. Pretty bad
   5. Very bad
   6. I don’t want to answer
10. Overall, how different do you feel from other females your age?
    1. Not different at all
    2. Only a little different
    3. Fairly different
    4. Very different
    5. Extremely different
    6. I don’t want to answer
11. As a child I felt my genitals were
    1. Like those of other girls
    2. Were different from other girls but were OK
    3. Were different from other girls and were not OK
    4. Were wrong or ugly
    5. I don’t want to answer
12. Now, how different are your genitals from other females your age?
    1. Not different at all
    2. Only a little different
    3. Fairly different
    4. Very different
    5. Extremely different
    6. I don’t want to answer
13. How do you feel about the appearance of your genitals?
    1. I like them as they are
    2. They are okay but I wish they looked better
    3. I am not happy with how they look
    4. I am very unhappy with how they look
    5. I don’t want to answer
14. In the last year, have you ever felt you wanted to be a man?
    1. Never
    2. Occasionally
    3. Sometimes
    4. Most of the time
    5. Always
    6. I don’t want to answer
15. I feel that I am a person of worth.
    1. Strongly agree
    2. Agree
    3. Disagree
    4. Strongly disagree
    5. I don’t want to answer
16. I am able to do things as well as most other people.
    1. Strongly agree
    2. Agree
    3. Disagree
    4. Strongly disagree
    5. I don’t want to answer
17. On the whole, I am satisfied with myself.
    1. Strongly agree
    2. Agree
    3. Disagree
    4. Strongly disagree
    5. I don’t want to answer
18. I usually take a positive attitude toward myself.
    1. Strongly agree
    2. Agree
    3. Disagree
    4. Strongly disagree
    5. I don’t want to answer
19. Regarding my sex organs, I have
    1. Strong positive feelings
    2. Moderate positive feelings
    3. No feeling one way or the other
    4. Moderate negative feelings
    5. Strong negative feelings
    6. I don’t want to answer
20. Regarding my figure or physique, I have
    1. Strong positive feelings
    2. Moderate positive feelings
    3. No feeling one way or the other
    4. Moderate negative feelings
    5. Strong negative feelings
    6. I don’t want to answer
21. How many female friends have you had contact with recently?
    1. Nine or more
    2. Five to eight
    3. Two to four
    4. One
    5. None
    6. I don’t want to answer
22. How many male friends have you had contact with recently?
    1. Nine or more
    2. Five to eight
    3. Two to four
    4. One
    5. None
    6. I don’t want to answer
23. How many times have you done things with friends socially in the last month?
    1. More than 5 times
    2. Three to four times
    3. Twice
    4. Once
    5. None
    6. I don’t want to answer
24. How often have you felt lonely in the past month?
    1. None
    2. A few times
    3. About half the time
    4. Most of the time
    5. Nearly always
    6. I don’t want to answer

The following section is about sexuality. Please remember that there are no right or wrong answers. Your answers will be very helpful in determining about sexual concerns among those with congenital adrenal hyperplasia.

1. How strong are the sensations that result from touching/stimulating your clitoris?
   1. Very good
   2. Good
   3. Adequate
   4. Not very good
   5. Poor
   6. Absent, there is no sensation
   7. I don’t want to answer
2. With stimulation of your clitoris and genital area, have you ever had an orgasm? (yes / no / don’t know)

If you have had orgasms, how many have you had

- 1. In the last year? ___
  2. In the last month? ___
  3. In the last week? ___
  4. Most days? _______
  5. I don’t want to answer

1. If you have not had an orgasm, can you tell why (circle as many as apply)?
   1. Am not interested in having one
   2. Have not had the time or place to try
   3. My genital area is not sensitive enough to keep trying
   4. I don’t feel I should try by myself
   5. I have not found anyone to stimulate me
2. In your mind, which gender would be your preferred sexual partner?
   1. Only males
   2. Mostly males
   3. Either males or females
   4. Mostly females
   5. Only females
   6. I don’t want to answer
3. What physical involvement have you had with a male? (circle all that apply)
   1. None
   2. Kissing
   3. Touching breasts (Fondling)
   4. Touching above and below the waist (Petting)
   5. Mouth to genitals (Oral sex)
   6. Genitals to genitals (Intercourse)
   7. I don’t want to answer
4. If you have had intercourse with a male, how old were you the first time?
   1. Younger than 14 years
   2. 14 – 15 years
   3. 16-17 years
   4. 18 – 19 years
   5. 20 years or older
   6. I don’t want to answer
5. What physical involvement have you had with a female? (circle all that apply)?
   1. None
   2. Kissing
   3. Touching breasts (Fondling)
   4. Touching above and below the waist (Petting)
   5. Mouth to genitals (Oral sex)
   6. Genitals to genitals
   7. I don’t want to answer
6. Concerning your genital function:
   1. Have you had an orgasm? yes / no
   2. Can you insert a tampon? yes / no
   3. Have you had intercourse with a male? yes / no
   4. If you have had intercourse, was having intercourse difficult? yes / no
   5. If you have had intercourse is it painful? yes / no
   6. If you have not had intercourse with a male, is this because you would prefer a female sexual partner? yes / no
7. Please write any further information or comments that you may have.

______________________________________________________________

______________________________________________________________

______________________________________________________________

May we contact you by telephone if any of your responses need to be clarified?
